# The categorical use of a continuous time representation

**DOI:** 10.1007/s00426-021-01553-y

**Published:** 2021-07-21

**Authors:** Alessia Beracci, Julio Santiago, Marco Fabbri

**Affiliations:** 1 Department of Psychology, University of Campania Luigi Vanvitelli, Viale Ellittico 31, 81100 Caserta, CE Italy; 2 Department of Experimental Psychology, University of Granada, Granada, Spain

## Abstract

The abstract concept of time is mentally represented as a spatially oriented line, with the past associated with the left space and the future associated with the right. Although the line is supposed to be continuous, most available evidence is also consistent with a categorical representation that only discriminates between past and future. The aim of the present study was to test the continuous or categorical nature of the mental timeline. Italian participants judged the temporal reference of 20 temporal expressions by pressing keys on either the left or the right. In Experiment 1 (*N* = 32), all words were presented at the center of the screen. In Experiment 2 (*N* = 32), each word was presented on the screen in a central, left, or right position. In Experiment 3 (*N* = 32), all text was mirror-reversed. In all experiments, participants were asked to place the 20 temporal expressions on a 10-cm line. The results showed a clear *Spatial–TEmporal Association of Response Codes (STEARC)* effect which did not vary in strength depending on the location of the temporal expressions on the line. However, there was also a clear Distance effect: latencies were slower for words that were closer to the present than further away. We conclude that the mental timeline is a continuous representation that can be used in a categorical way when an explicit past vs. future discrimination is required by the task.

## Introduction

There is a growing consensus that abstract concepts such as numbers and time have a spatial mental representation. According to the literature, both numbers and time are represented along a laterally oriented line (Bonato et al., 2012). Thus, these lines are often referred to as the mental number line (MNL) and mental time line (MTL). One of the most important challenges in embodied and grounded cognition theories is to understand how abstract concepts (such as time, numbers, emotions, etc.) are acquired, represented, and used, and it is recognized that language plays an important role (Borghi, 2020). Language can work as both an inner and a social tool that influences the mental representation of abstract concepts through spatial mappings (Borghi & Setti, 2017). In addition, an investigation into the nature of the time line could shed light on how this representation indicates how we interact with the outside world, how (and why) we associate temporal elements with space, and, finally, how we store memories in long-term memory, by rebuilding the mapping of life events.

The main supporting evidence regarding the MNL is the *Spatial-Numerical Association of Response Codes*, or SNARC effect (b; Dehaene et al., 1993; Hubbard et al., 2005; Nuerk et al., 2005a; Schwarz & Keus, 2004), in which smaller numbers are responded to faster with left-sided responses, and relatively, larger numbers are responded to faster with right-sided responses.

Likewise, in the temporal domain, an analogous effect to the SNARC effect has been found, for which Ishihara et al. (2008) coined the term *Spatial–TEmporal Association of Response Codes*, or STEARC effect. This effect has been found with three different strategies that are used to convey temporal information. The first strategy uses words that refer to either the past or the future (Santiago et al., 2007; Torralbo et al., 2006). The second strategy uses stimuli that vary in actual duration (Fabbri et al., 2013a, b; Ishihara et al., 2008; Vallesi et al., 2008). A third strategy uses events (presented by means of pictures) that can be construed as having a particular temporal location within a wider sequence (Fuhrman & Boroditsky, 2010; Santiago et al., 2010). All three strategies have provided converging results that support a left-to-right spatial mapping along the MTL: shorter durations, expressions referring to the past, and prior events were responded to faster with left-sided responses, while longer durations, expressions referring to the future, and subsequent events were responded to faster with right-sided responses. Although Ishihara et al. (2008) coined the term “STEARC effect” specifically in the context of the second strategy, we contend that the effect revealed by the three strategies is actually one and the same, and we will use the term generally hereafter.

The similarities between the SNARC and the STEARC effects go further than the intuitively obvious analogy between ordered numerical and temporal series that run from left to right (see Bonato et al., (2012), for a detailed comparison). An important argument supporting the underlying representations having a great deal in common is the relationship of these effects with reading and writing direction. In their seminal report regarding the SNARC effect, Dehaene et al. (1993) reported that the effect is reduced in participants who read from right to left. Later, Zebian (2005) reported a reversed SNARC in Arabic monoliterates and Arabic-English biliterates; and Shaki et al. (2009) found the same result in Palestinians. Evidence for a causal link was provided by Fischer et al. (2010), who changed the SNARC by manipulating the location of smaller and larger numbers on the page.

Similarly, the STEARC effect has also been shown to depend on reading and writing direction. Ouellet et al. (2010) found a reversed STEARC effect in Hebrew readers using words with a temporal reference, and Fuhrman and Boroditsky (2010) reported the same result using pictures of sequential events. Vallesi et al. (2014) found that the STEARC effect disappeared (although it was not reverted) in Hebrew readers. Casasanto and Bottini (2014) were able to revert the STEARC effect by asking their participants to judge the temporal reference of mirror-reversed expressions in Dutch, thus proving a causal link between reading direction and the directionality of the STEARC. Thus, both the MNL and the MTL seem to arise from directional experiences that correlate with the direction of the habitual script, suggesting a cultural origin.

A non-cultural account of congruency effects that might be applicable to the SNARC and the STEARC effects was proposed by Proctor and Cho (2006), and was based on the idea of polarity correspondence. For a variety of binary classification tasks, participants code the stimulus alternatives and response alternatives as (+) polarity and (−) polarity, and response selection is faster when the polarities correspond than when they do not. Indeed, the SNARC and the STEARC effect could be due to correspondence in magnitude polarity [large number, long durations, “after”, future (+); small number, short durations, “before”, past (−)] and response polarity [right (+); left (−)]. However, the polarity correspondence account of the SNARC effect was explicitly put to the test by Santiago and Lakens (2015), who found it unsupported by the data.

Additional evidence supporting the similarity between the SNARC and the STEARC effects was provided by Fabbri et al. (2012), who used a procedure in which time, numbers, and space were all task-relevant, and observed a triple interaction between all the elements. Additionally, Anelli et al. (2018), when examining the directionality of the MTL with a bimodal response setting, showed that the STEARC effect emerged at the stage of response selection (Anelli et al., 2018; for additional findings see Vallesi et al., (2008, 2011)), in agreement with the previous findings concerning the SNARC effect (Gevers et al., 2006; Keus & Schwarz, 2005). All the previous results showing how the SNARC and STEARC effects share similar features suggest that the mental representations of numbers and time have a lot in common, being constituted in both cases by a mental line that runs along the lateral axis.

It is intrinsic that a mental line has an analogical and continuous nature. However, this assumption has not been systematically verified. For example, Gevers et al. (2006) demonstrated that the SNARC effect (and consequently the spatial characteristics of the MNL) was categorical or continuous depending on task instructions. The authors observed that the size of the SNARC effect (the difference between the latency of right- and left-hand responses) varied continuously with number magnitude in a parity judgment task, but was oblivious to number size in a magnitude comparison task, suggesting an underlying categorical representation in the latter (see Fig. 2A vs. B in Gevers et al., (2006), p. 34; Fig. 3A vs. B, in Gevers et al., (2006), p. 35). The authors provided a general framework with which to interpret the SNARC effect, postulating the existence of two routes of activation during the processing of numbers: a relatively fast route that automatically codes for the location of the numeric stimulus on the line and a relatively slow route that is dependent on the task instruction and provides the mapping of the relevant attribute to the required response. When both routes converge on the same spatial response code (a congruent condition), the response can be relatively fast and accurate. When both routes converge on opposing response codes (an incongruent condition), reaction times are slower, and errors are more frequent. The evidence from the number domain is thus maximally consistent with an underlying continuous representation that can be used categorically when the task imposes explicit magnitude comparisons with a criterion.

Can we extrapolate this account to the temporal domain? In order to do so, the STEARC effect should be categorical in explicit tasks and continuous in implicit tasks. The available findings are inconclusive. Is the STEARC effect continuous in implicit time tasks? The main issue in answering this question is that it is unclear whether, and how, it is possible to find a STEARC effect in implicit tasks at all. Ulrich and collaborators (Ulrich & Maienborn, 2010; Ulrich et al., 2012; Maienborn et al., 2015) asked participants to decide whether past and future-related sentences were sensible or nonsensical by moving a lever. In this implicit task, they failed to observe the STEARC effect at all, but the effect was clearly present when the task was to judge the temporal reference of the sentence. The authors reasoned that, when time is task-irrelevant, either the MTL is not automatically activated by the linguistic information or its activation is too weak to be detected.

However, there are studies which have succeeded in observing an STEARC effect in an implicit task. Lakens et al. (2011) asked participants to place eight past and eight future-related expressions on a horizontal line. The expressions varied in their temporal distance from the present (from “past” through “the day before yesterday”, “a moment ago”, “immediately”, “the day after tomorrow”, to “future”). The results showed that the words were positioned at different locations on the line following a left-to-right progression that agreed with their temporal reference, suggesting that their meanings are represented along a spatial continuum. In a subsequent experiment, the expressions were presented auditorily and were intermixed with words that were unrelated to time (e.g., “table” or “glass”). Participants were asked to judge which ear was presented with the louder stimulus. Crucially, time-related expressions were all presented with the same volume. The results showed that future-related words were judged to be louder in the right ear more often than past-related words. Furthermore, the visual-spatial ordering of the expressions obtained in Experiment 1 was linearly related to the probability of judging an expression as louder in the right ear. The authors also tested the relationship using a categorical predictor (past vs. future), but a significant fit failed to emerge, suggesting that the pattern was linear. However, this study has one important caveat: the temporal range of the expressions was small. Besides the words “past” and “future”, which do not refer to any specific temporal distance, the remaining expressions ranged from the day before yesterday to the day after tomorrow. Moreover, the agreement between participants in locating some of the expressions may have been low (e.g., “been”, “coming”). These factors may underlie the fact that the observed linear relationship, though significant, was small (*r*^2^ = 0.30), and may elicit doubts about its replicability.

Because of the strong relationship between sequential order and temporal order, studies using sequences of ordered elements are also relevant to the present discussion. However, they also suffer from problems that make them inconclusive. Gevers et al., (2003, 2004) presented months, letters of the alphabet, and days of the week to be compared with a central reference (explicit task), as well as in implicit, order-irrelevant tasks (e.g., say whether the month name contains the letter “R”). They observed STEARC effects in both the explicit and implicit tasks. Inspection of the figures suggests a categorical pattern in the explicit-order judgment tasks and a continuous pattern in the implicit tasks. However, they did not contrast a categorical versus a continuous predictor, and therefore, it is uncertain whether the observed pattern should be considered continuous or categorical.

Is the STEARC effect categorical in explicit time judgment tasks? Available evidence does not provide a clear answer to this question either. Vallesi et al. (2008) asked their participants to judge the duration of a visual stimulus varying from 0.5 to 3.5 s as smaller or larger than a criterium (2 s), and compared the degree of fit obtained by a continuous and a categorical predictor of the size of the STEARC effect. The categorical predictor provided a better fit than the continuous predictor. However, Ding et al. (2015) found some evidence of a continuous pattern while exploring the symmetry of the STEARC effect toward the past and the future. They compared the size of the effect when expressions that referred to a close (a day) versus a far (a year) distance in the past or the future were judged. They showed that the STEARC effect was greater for temporally distant than for closer events, suggesting a continuous underlying representation. However, this study also has many caveats: first, only past, but not future distant events showed a greater STEARC effect; and second, there was no explicit test for a continuous vs. categorical STEARC effect. Also inconclusive is the study by Santiago et al. (2010) which used an explicit-order judgment task. The authors asked participants to watch movie clips or picture sequences depicting everyday events (e.g., getting up and making breakfast). Participants then had to classify clip frames or pictures as belonging to the first or second half of the story by means of left or right key presses (there was no implicit task). In this case, visual inspection of the figures suggests a categorical pattern, but the authors did not expressly test for this by comparing a categorical vs. a continuous predictor.

It is important to note that in all the studies that tested for a Distance effect, it was in the explicit tasks that the effect was found: responses were slower for items that were closer to the reference (Gevers et al., 2003, 2004; Santiago et al., 2010). Since Moyer and Landauer (1967) originally reported that comparing closer numbers is more difficult than comparing distant numbers, the Distance effect has been considered the hallmark of a continuous representation of numbers (see Dehaene (1992), for a review; Dehaene et al., 1993; Fias, 2001; Fias et al., 1996). Thus, this effect would suggest a continuous underlying representation of time in explicit tasks. However, the data are again unclear: while Santiago et al. (2010) found a symmetrical Distance effect, Gevers et al. (2004) found it only on one side (Gevers et al., 2004), and it was also affected by unexplained interactions (Gevers et al., 2003).

In summary, in terms of the existence of a continuous or categorical representation underlying the STEARC effect in explicit and implicit temporal tasks, the evidence is scarce, mixed, and unclear. Because of the difficulty of finding the STEARC effect in implicit tasks, we focused on an explicit task. The main aim of our study was to provide a conclusive test of (1) whether the STEARC effect is continuous or categorical, and (2) whether there is a Distance effect when an explicit temporal judgment is required. Thus, in three experiments, we requested participants to perform a STEARC explicit temporal task in which they had to categorize, as past or future, 20 Italian temporal expressions that referred to time points varying from the distant past to the distant future. To make sure that the meanings of the temporal terms were distributed more or less continuously over space and to estimate their spatial location with precision, after the STEARC task, we asked the same participants to place them on a line. This also allowed us to use the position of each term on the line as reported by each individual to predict both the Distance effect (the effect of spatial position on average reaction time) and the STEARC effect (the effect of spatial position on the difference in reaction time between the right and left responses) of that individual. This allowed us to reach general conclusions regarding the relationship between space and time at the same time as we controlled for idiosyncratic patterns. More importantly, we followed the logic used by Gevers et al. (2006), Vallesi et al. (2008), and Lakens et al. (2011) to directly test for a continuous versus a categorical pattern in the STEARC effect by contrasting the goodness of fit of a continuous predictor with a categorical predictor. The three experiments were close replications that varied only in aspects of stimulus presentation: central presentation in Experiment 1, central and lateral presentation in Experiment 2, and mirror-reversed central and lateral presentation in Experiment 3. With reference to the study by Santiago et al. (2011), we reasoned that the degree of saliency of the lateral axis might affect how this axis is used to perform the task. In all experiments, the lateral axis is task-relevant, because it is used to guide manual responses. In Experiment 2, we expected its saliency to increase due to the presence of a left–right contrast of stimulus presentation on the screen. In Experiment 3, we used mirror-reversed stimuli following the work by Casasanto and Bottini (2014). We expected this manipulation to reverse the STEARC effect, but our main goal was to push the saliency of the left–right axis to the extreme, in the hope of capturing a continuous STEARC effect. Thus, the decision to perform three different experiments with different degrees of saliency of the spatial left–right axis was made to provide a strong test of continuous vs. categorical nature of the MTL in explicit tasks.

## Methods

### Participants

The studies reported here were approved by the Ethical Committee of the University of Caserta. All participants were Italian university students, who took part in exchange for course credit, had normal or corrected-to-normal vision, provided written informed consent, and filled in a demographic questionnaire as well as the Edinburgh Handedness Inventory (EHI; Oldfield, 1971). Experiment 1 included 32 students (29 females; mean age = 24.44 years; SD = 2.00 years). According to the EHI scores, 28 were right-handed (*M* = 92.44; SD = 13.42) and 4 were left-handed (*M* = − 75.00; SD = 30.62). Experiment 2 included a separate group of 32 students (30 females; mean age = 23.91 years; SD = 1.65 years; 30 were right-handers, *M* = 89.73; SD = 17.72; and 2 left-handers, *M* = − 94.74; SD = 7.44). In Experiment 3, there was a new sample of 32 students (27 females and 5 males; mean age = 24.13 years; SD = 1.48 years; 26 were right-handed, *M* = 85.37; SD = 14.82; and 6 were left-handed, *M* = − 82.48; SD = 15.95).

### Materials

All experiments reported hereafter used the same set of materials. The stimuli consisted of 20 Italian temporal expressions, ten referring to the past ("anticamente", formerly; "tempo fa", long ago; "in passato", in the past; "una volta", once; "precedentemente", previously; "l’altro ieri", the day before yesterday, "l’altro giorno", the other day; "prima", before; "ieri", yesterday; "recentemente", recently) and 10 referring to the future ("dopo", after; "tra poco", in a short while; "presto", soon "conseguentemente", subsequently; "domani", tomorrow; "in seguito", later; "dopodomani", the day after tomorrow; "successivamente", thereafter; "prossimamente", next; "in futuro", in the future). Note that the temporal reference of the English translations may not match exactly the reference in Italian.

### Procedure

All stimuli were presented in white font on a black laptop computer screen. The display had a resolution of 1072 × 960 pixels and a refresh rate of 72 Hz. Participants sat at a viewing distance of 55 cm and were individually tested in a quiet room. Stimulus presentation and data collection were controlled using E-Prime 2.0 (Schneider et al., 2002).

**Fig. 1:**
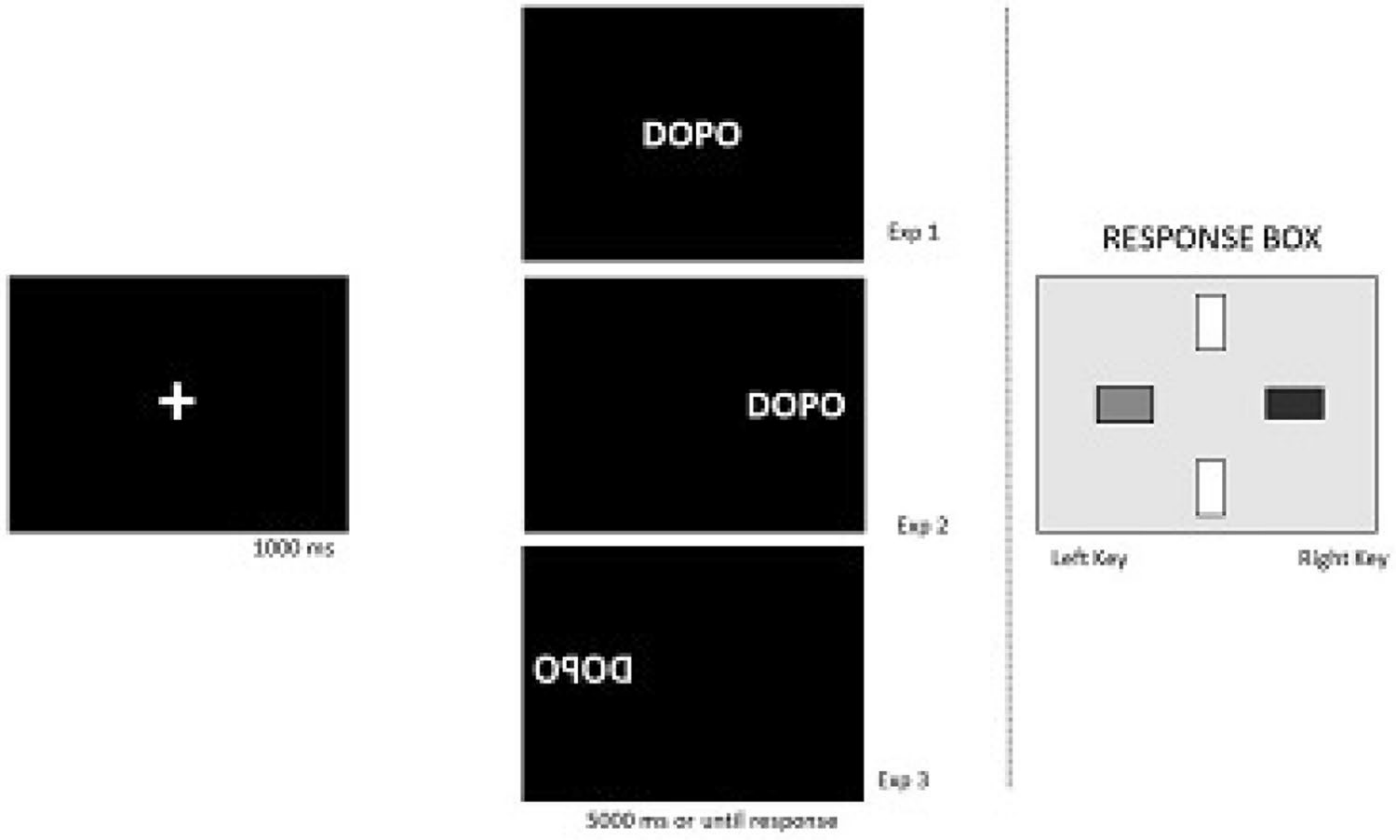
Example of trial sequences of the temporal judgment task

The experimental session had two parts, for a total duration of approximately 20 min. In the first part, participants were required to categorize the target temporal expressions as referring to the past or to the future by means of a yellow key on the left and a blue key on the right of a response box (Cedrus RB- × 40 Response Pads) that was connected to the laptop. In each trial, a fixation cross (+) symbol was presented first at the center of the screen for 1000 ms, followed by a temporal expression for 5000 ms or until a response was recorded. All target stimuli were presented in two separate blocks with different time–key mappings. In one block, the left key was pressed to categorize the target as past, and the right key to categorize the target as future (see Fig. 1). The other block had the reverse mapping. The order of blocks was counterbalanced over participants. Each block comprised a total of 180 trials (nine repetitions of each target expression) in a random order. Thus, altogether, the participants judged 360 trials. Before the test, a 10-trial training block was run. The training phase could be repeated if requested by the participant. After each block, the participant was allowed to take a short break.

**Fig. 2:**
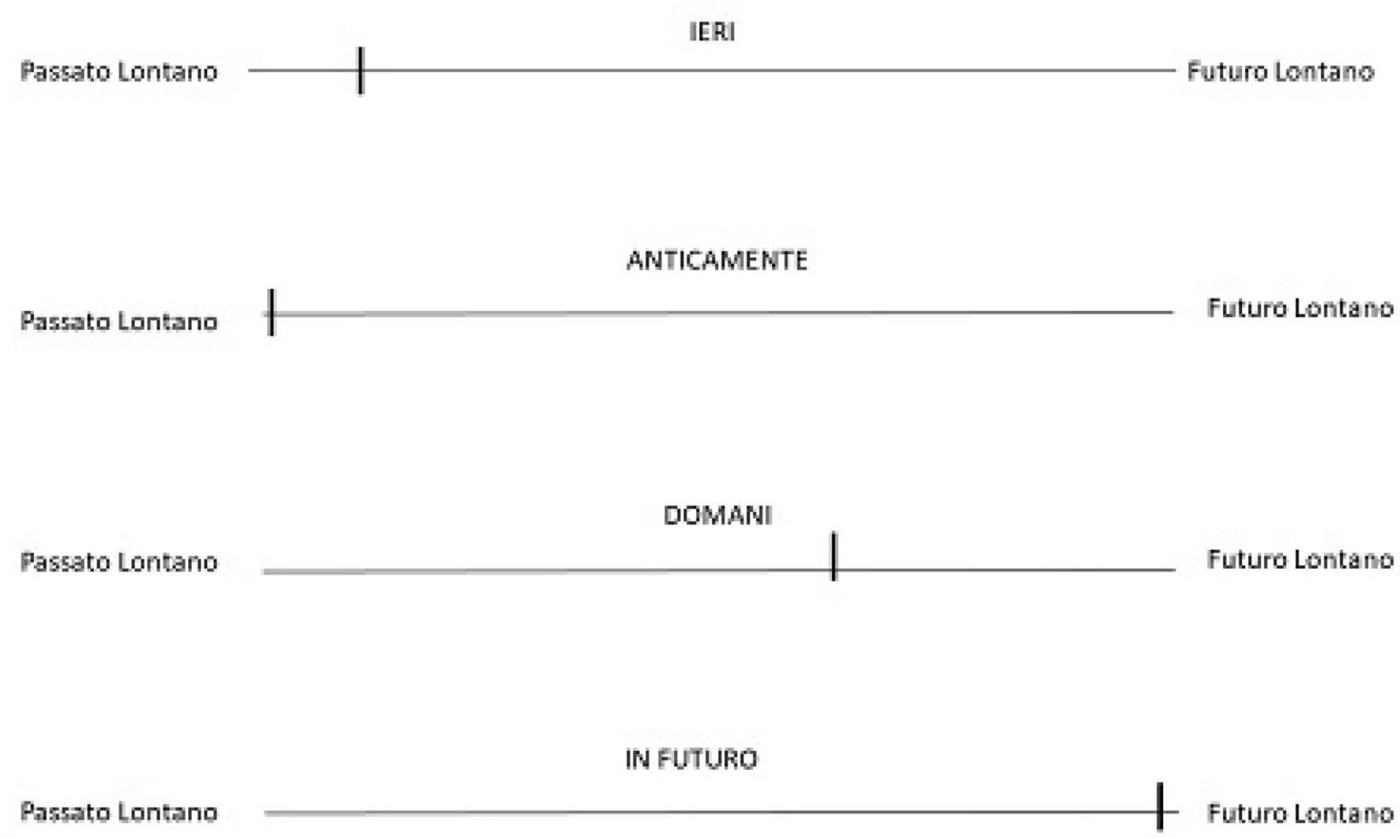
Example of several trials in the time-to-position task. Only one stimulus and line was presented in each trial

After the time judgment task, participants were requested to perform a paper-and-pencil Time-to-Position task (see Fig. 2). They were presented with each of the 20 temporal expressions written above a 10-cm long line that was flanked by the labels “*passato lontano*” (“distant past”) on the left and “*futuro lontano*” (“far future”) on the right. Only one expression was presented in each trial, in a randomized order, and participants could not see their responses to prior expressions. They then had to decide where the temporal meaning of the expression should be positioned on the line by making a vertical mark with a pencil (see Fabbri and Guarini (2016), and Fabbri and Natale (2016), for a similar procedure with numbers).

**Fig. 3:**
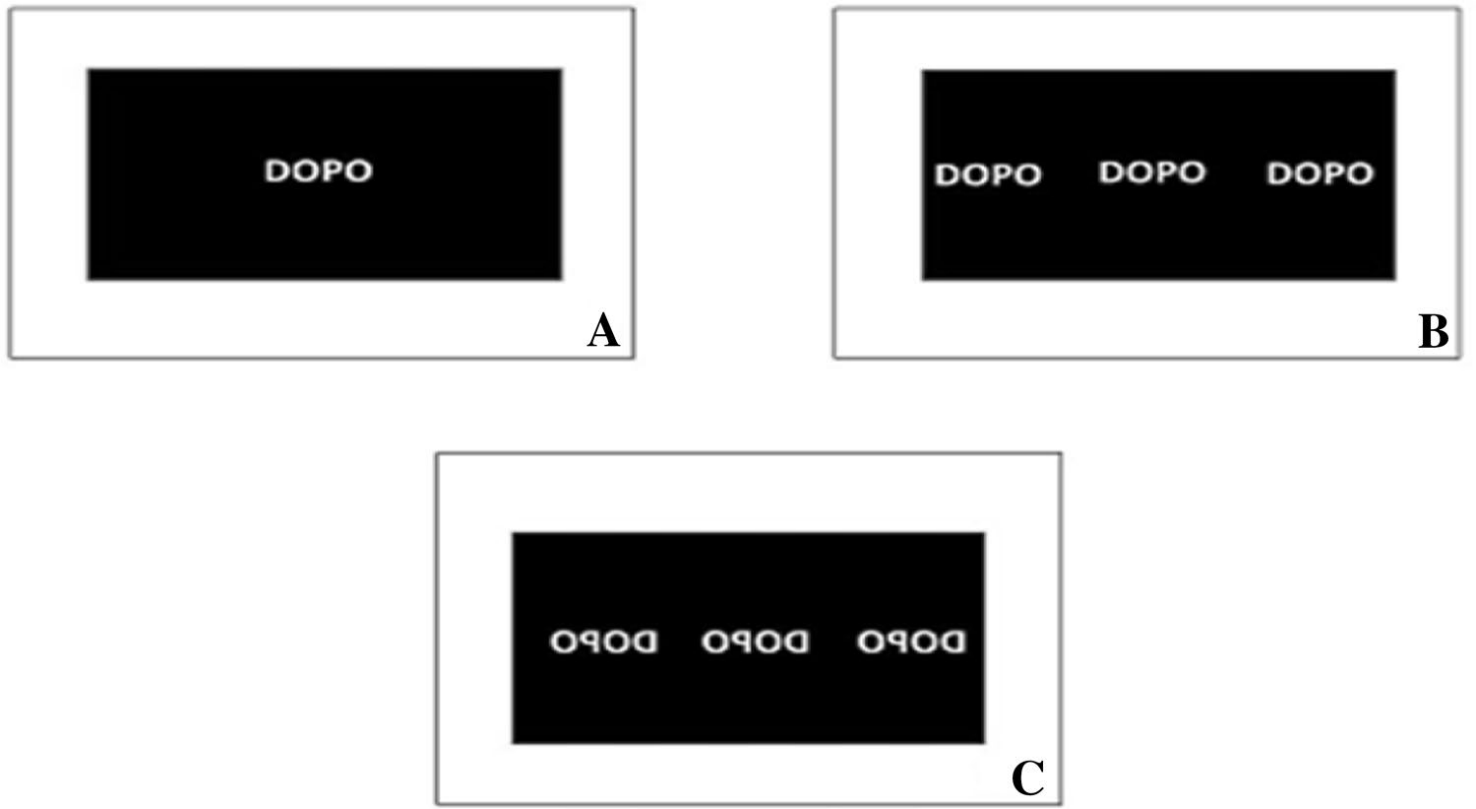
**a** Example of stimulus presentation in Experiment 1; **b** locations of stimuli presentation in Experiment 2; **c** locations of stimuli presentation in Experiment 3. Note that in experiments 2 and 3, only one expression was presented on the screen in each trial

The only difference between experiments regarded the presentation of the target stimuli (see Fig. 3). In Experiment 1, all target expressions were presented centrally on the screen. In Experiment 2, expressions were presented either at the center, on the left or on the right of the screen. In Experiment 3, expressions were mirror-reversed and presented at the same three locations as in Experiment 2.

## Data analysis

To verify the presence of an STEARC and a Distance effect and to assess whether the former takes a continuous or categorical form, data were analyzed as follows.

First, we analyzed the Time-to-Position Task, to make sure that the reference of the temporal expressions spreads more or less homogeneously over the space from the distant past to the distant future. As in the previous studies (Ebersbach et al., 2008; Fabbri & Guarini, 2016; Fabbri & Natale, 2016; Siegler & Opfer, 2003; White & Bull, 2008), we measured (in mm), for each participant and each temporal expression, the distance from the left side of the line to the subjective mark positioned on the line. To put these measures on a common metric, we computed *z* scores for each participant. This standardized score indicated how far the subjective position of each expression on the line was from the subjective center (average position of all expressions) in standard deviation units.

Second, we focused on reaction times (RTs) in correct trials (94.98% of total trials) and analyzed them by means of mixed models (LMM) using the package lme4 (Bates et al., 2015) in R version 3.6.1 (R Core Team, 2018). The models used sum contrasts. Categorical predictors were dummy coded and centered. As in the studies by Barr et al. (2013) and Barr (2013), we started by specifying the maximally complex model and then simplified it until the convergence problems were resolved, and then until the simplest model was found that did not lose goodness of fit (b; Bates et al., 2015). To compare models, we used likelihood tests as implemented in the anova() function of the package LmerTest (Kuznetsova et al., 2017). The final choices of parametric (linear) models were interpreted by means of the ANOVA tables and p values provided by the anova() function of LmerTest using the Sattherwaite method. Generalized models were interpreted using the Anova() function of the package car (Fox & Weisberg, 2019). Whenever we found convergence problems, we re-ran the analysis using the bobyqa optimizer, which sometimes succeeded (using a different optimizer does not change the results of a model, but may sometimes alleviate convergence problems).

RTs smaller than 200 ms or larger than 2.5 SDs above the participant’s mean were discarded as outliers (3.25% of correct trials). To test whether the STEARC effect takes a continuous or categorical form, we first averaged the RT for each hand, item, and participant. Then, we subtracted the RT of the left hand from the RT of the right hand. The resulting distribution of differential RTs showed no asymmetry, lending validity to the use of parametric approaches of analysis (i.e., assuming a Gaussian distribution). Differential RTs were predicted from the fixed factors Experiment (1–3), time (past vs. future), and Distance in the Time-to-Position Task. We followed two parallel analytical approaches: one started with the continuous predictor, found the simplest model, and then added the categorical predictor; another started with the categorical predictor, found the simplest model, and then added the continuous predictor. In the final models, we could thus compare which (the continuous or the categorical) predictor provided the best fit.

To assess the Distance effect, for each participant and item, we computed the average RT of both left and right responses. We also computed the absolute values of the standardized distances from the subjective center, so that they served as a non-directional measure of distance from the center. The effect of Absolute Distance was then assessed together with the effects of categorical Time (past vs. future), Experiment (1–3), and their interactions. An interaction between Absolute Distance and Time would reveal asymmetrical Distance effects toward the past and the future. Mean RTs showed a skewed distribution, so we used the strategy proposed by Lo and Andrews (2015): using generalized mixed models, we compared a model that assumed a Gamma distribution with another assuming an Inverse Gaussian distribution, and retained the best model as the starting point of the analysis.

All data and analysis scripts are available for download via the following Open Science Framework repository: https://osf.io/7jckp/quickfiles.

## Results

### Time-to-position task

**Fig. 4:**
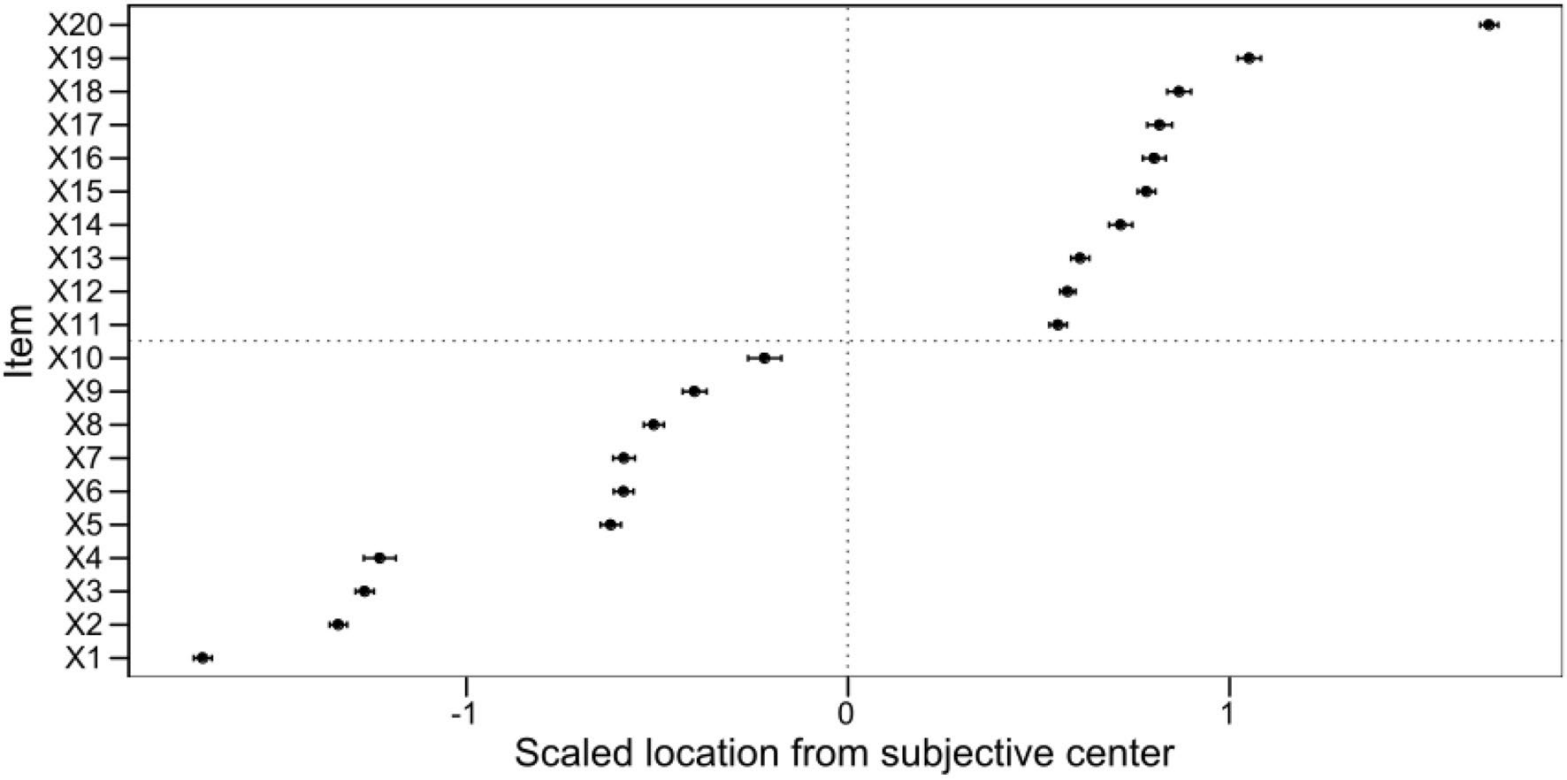
Distance from the subjective center in SD units for each temporal expression in the time-to-position task. Error bars show SEM. Items: X1 = Anticamente, X2 = Tempo fa, X3 = In passato, X4 = Una volta, X5 = Precedentemente, X6 = L’altro ieri, X7 = L’altro giorno, X8 = Prima, X9 = Ieri, X10 = Recentemente, X11 = Dopo, X12 = Tra poco, X13 = Presto, X14 = Conseguentemente, X15 = Domani, X16 = In seguito, X17 = Dopodomani, X18 = Successivamente, X19 = Prossimamente, and X20 = In futuro

As shown in Fig. 4, the standardized distances to the subjective center in the Time-to-Position task revealed that the 20 temporal terms spanned a wide spatial interval, always keeping to the expected order. Thus, Italian speakers placed the 20 temporal expressions in an ordered pattern from left to right along a linear (time) line. Although the selected temporal expressions did not fall at regularly spaced intervals, their number and dispersion support their use as a continuous predictor of reaction times in the experimental tasks.

### Continuous vs. categorical STEARC effect

In the first analytical approach, we started using the continuous predictor (see Table 1 for a description of the sequence of analytical steps and their results). The random term in the maximal model included random intercepts per participant as well as random slopes of standardized Distance over participants. The fixed effects were Experiment (1–3), Distance, and their interaction. The dependent variable was the right-hand RT minus left-hand RT for each item and participant. The maximal model failed to converge. Inspection of the results suggested that the convergence problems arose because of variability in random intercepts that was too small (as the dependent variable was the difference in latencies between the two hands, it showed a size close to zero and a very small variability). Therefore, we removed participant intercepts from the random term, keeping the random slopes of Distance. This model did converge and revealed a main effect of Distance but no effect of Experiment or interaction with Distance. We then searched for the simplest model without loss of fit. A model keeping only the Distance factor did not have a significantly different goodness of fit (*χ*^2^(4) = 0.43, *p* = 0.98), so we removed Experiment and the Experiment × Distance interaction. In this simplest model, Distance had a very clear effect (*F*(1,94.94) = 29.46, *p* < 0.001). Therefore, in agreement with our expectations, there was a clear STEARC effect. In contrast to expectations, the STEARC effect did not change over experiments, even when all the materials were mirror-reversed in Experiment 3. Of central relevance to the question of interest, in the simplest model, we then introduced the categorical predictor Time (past vs. future). This change produced an important increase in overall goodness of fit (*χ*^2^(1) = 77.57, *p* < 0.001). Moreover, the categorical predictor Time became the only factor responsible for the fit, as it showed a significant main effect (*F*(1,1819.6) = 79.15, *p* < 0.001), while the continuous predictor Distance became non-significant (*F*(1,194.2) = 0.15, *p* = 0.70). To provide a final check, we removed the continuous predictor from the model. The goodness of fit of the resulting model, including only the categorical predictor Time, did not differ from the model that included both predictors (*χ*^2^(1) = 0.15, *p* = 0.70).

**Table 1:** Comparisons of fit models

Model equation	Fixed effects	Model fit				
		Df	AIC	BIC	LL	Deviance
RminusL ~ Exp + stmm + Exp:stmm + (0 + stmm|SubjectID)	Experiment + distance + experiment × distance	8	26,024	26,068	− 13,004	26,608
RminusL ~ stmm + (0 + stmm|SubjectID)	Distance	4	26,016	26,039	− 13,004	26,608
RminusL ~ stmm + Time + stmm:Time + (0 + stmm|SubjectID)	Distance + time + distance × time	5	25,941	25,969	− 12,965	25,931
RminusL ~ Time + (0 + stmm|SubjectID)	Time	4	25,939	25,961	− 12,966	25,391

*AIC* Aikake Information Criterion, *BIC* Bayesian Information Criterion, *LL* LogLikelihood, *Df* degrees of freedom, *RminusL* right hand (RTs) minus left hand (RTs), *Exp* Experiment (1–3), *stmm* distance, *Time* time (past vs. future), *1 + Time|SubjectID* random intercepts and random slopes of Time over participants

In the second analytical approach, we started using the categorical predictor Time (see Table 2). The random term of the maximal model included random intercepts for participants and random slopes over Time. The fixed effects included Time, Experiment, and their interaction. The maximal model converged and revealed only a main effect of Time. We then removed Experiment and the interaction Time x Experiment from the model and compared the resulting simplest model with the maximal model: the goodness of fit did not change (*χ*^2^(4) = 0.53, *p* = 0.97). The effect of the categorical predictor Time was very clear (*F*(1,94.96) = 30.29, *p* < 0.001). We then added the continuous Distance predictor to the simplest model, but it did not improve fit (*χ*^2^(1) = 0.83, *p* = 0.36).

**Table 2:** Comparisons of fit models

Model equation	Fixed effects	Model fit				
		Df	AIC	BIC	LL	Deviance
RminusL ~ Exp + Time + Exp:Time + (1 + Time|SubjectID)	Experiment + time + experiment × time	10	25,723	25,779	− 12,852	25,703
RminusL ~ Time + (1 + Time|SubjectID)	Time	6	25,716	25,749	− 12,852	25,704
RminusL ~ Time + stmm + (1 + Time|SubjectID)	Time + distance	7	25,717	25,756	− 12,852	25,703

*AIC* Aikake Information Criterion, *BIC* Bayesian Information Criterion, *LL* LogLikelihood, *Df* degrees of freedom, *RminusL* right hand (RTs) minus left hand (RTs), *Exp* Experiment (1–3), *stmm* distance, *Time* time (past vs. future), *1 + Time|SubjectID* random intercepts and random slopes of time over participants

**Fig. 5:**
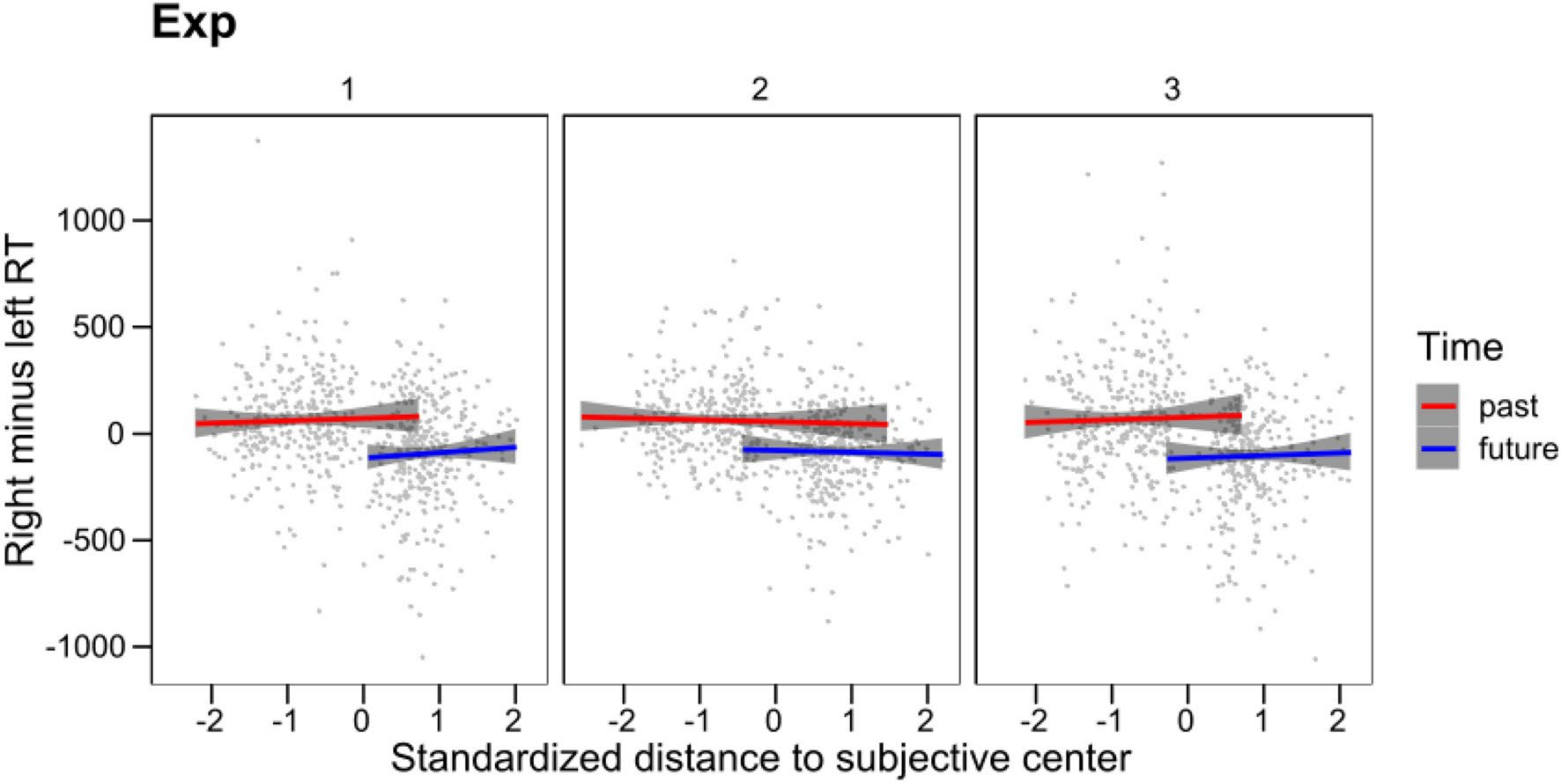
Differential RTs (average RT of the right hand for each participant and expression minus the average of those produced with the left hand) for the past and future expressions. Lines represent the best fitting linear model within each temporal category. The gray areas represent the 95% confidence interval

To provide a final test of the categorical nature of the STEARC effect, we ran independent analyses of the effect of the continuous predictor Distance on each Time condition (past vs. future; see Table 3). In the Past condition, the maximal model included the effects of Distance, Experiment, and their interaction, random intercepts for participants and random slopes of Distance over participants. The model converged and revealed no significant findings. We simplified the model, leaving only the fixed effect of Distance. The resulting simplest model did not differ from the maximal model in goodness of fit (*χ*^2^(4) = 0.15, *p* = 0.997). In this model, the effect of Distance was also non-significant (*F*(1,79.94) = 0.46, *p* = 0.50). In the Future condition, the maximal model converged and also showed no significant findings. The simplest model, including only the fixed effect of Distance, produced a singular fit, so we also simplified the random term by removing the participant intercepts, which solved the problem. To allow a fair comparison of the goodness of fit of the maximal and the simplest models, we also removed the participant intercepts from the maximal model (which did not change the null findings). The simplest model showed the same goodness of fit as the latter maximal model (*χ*^2^(4) = 0.71, *p* = 0.95). In the simplest model, Distance failed to make any significant contribution (*F*(1,292.63) = 0.21, *p* = 0.65). Therefore, when analyzed only within the range of past or future expressions, the continuous predictor Distance failed to have any effect on differential RTs (see Fig. 5).

**Table 3:** Comparisons of fit models

Model equation	Fixed effects	Model fit				
		Df	AIC	BIC	LL	Deviance
Past						
RminusL ~ Exp + stmm + Exp:stmm + (1 + stmm|SubjectID)	Experiment + distance + experiment distance	10	12,958	13,007	− 6469.0	12,938
RminusL ~ stmm + (1 + stmm|SubjectID)	Distance	6	12,950	12,979	− 6469.1	12,938
Future						
RminusL ~ Exp + stmm + Exp:stmm + (1 + stmm|SubjectID)	Experiment + distance + experiment × distance	10	12,874	12,923	− 6427.3	12,844
RminusL ~ stmm + (1 + stmm|SubjectID)	Distance	6	12,867	12,896	− 6427.7	12,855
RminusL ~ stmm + (0 + stmm|SubjectID)	Distance	4	12,932	12,951	− 6461.8	12,924
RminusL ~ Exp + stmm + Exp:stmm + (0 + stmm|SubjectID)	Experiment + distance + experiment × distance	8	12,939	12,923	− 6461.4	12,923

*AIC* Aikake Information Criterion, *BIC* Bayesian Information Criterion, *LL* LogLikelihood, *Df* degrees of freedom, *RminusL* right hand (RTs) minus left hand (RTs), *Exp* Experiment (1–3), *stmm* distance, *Time* time (past vs. future), *0 + stmm|SubjectID* no random intercept and random slopes of Distance over participants, *1 + stmm|SubjectID* random intercepts and random slopes of Distance over participants

### Distance effect

**Fig. 6:**
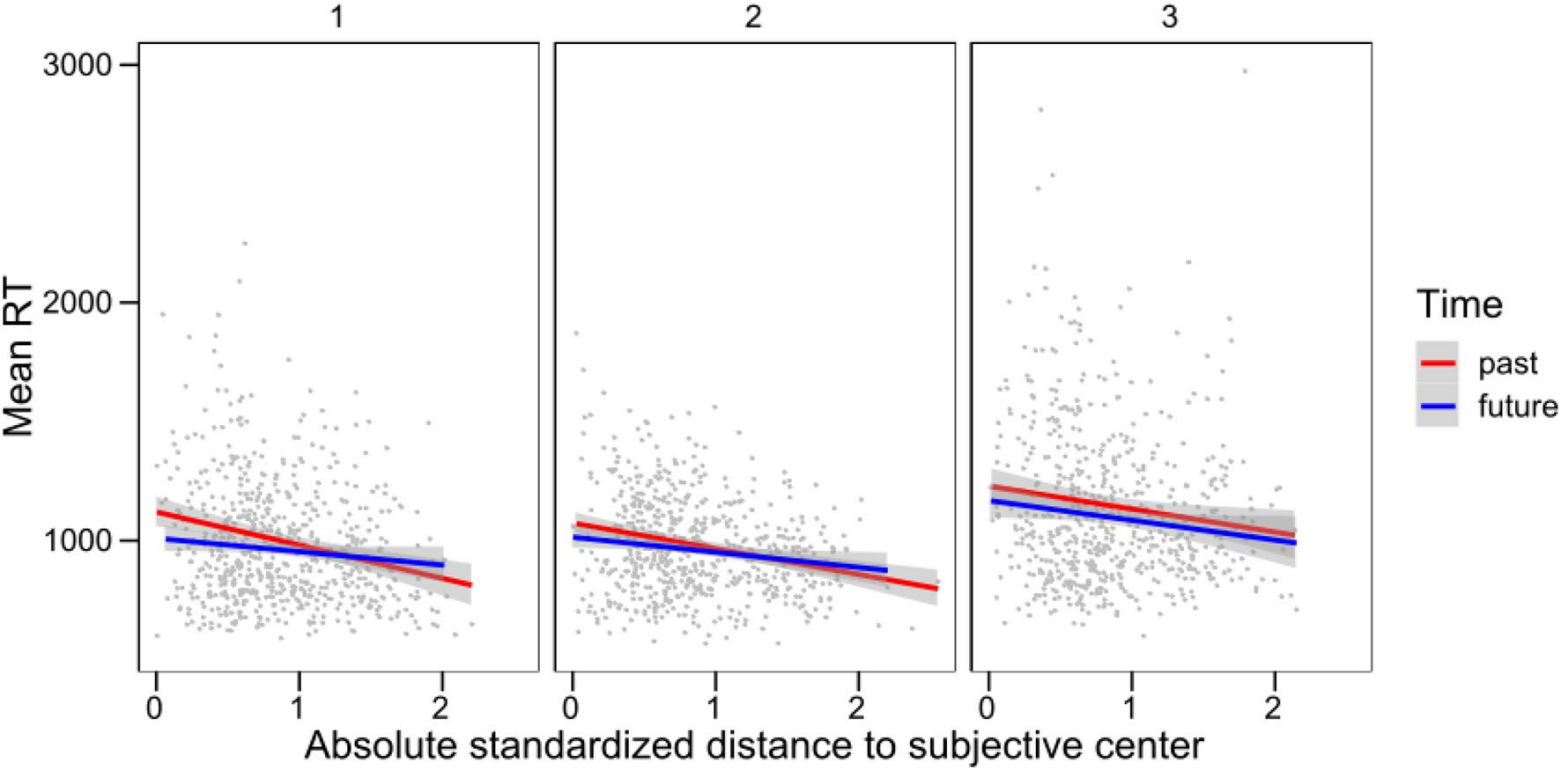
Average RTs for each participant and expression for the past and future expressions. Lines represent the best fitting linear model within each temporal category. The gray areas represent the 95% confidence interval

The analysis of the Distance effect used the factor Absolute Distance (see above) to produce a non-directional estimation of distance from the subjective center (see Table 4). The fixed effects in the maximal model included the factors Absolute Distance, the categorical factor Time (past vs. future), Experiment, and all their interactions. The random term included participant intercepts, and the random slopes over participants of Absolute Distance, Time, and their interaction. As in the study by Lo and Andrews (2015), we tried both a model that assumed an Inverse Gaussian distribution and one that showed a Gamma distribution. The former model failed to converge, so we continued the analysis using the latter. The maximal model revealed main effects of experiment, absolute distance, and time, as well as an absolute distance × time interaction. We then simplified the model by removing all the non-significant interactions. The resulting simplest model did not change goodness of fit (*χ*^2^(4) = 0.71, *p* = 0.95). Any further simplifications reduced the predictive ability of the model. The simplest model showed clear main effects of Experiment (*χ*^2^(2) = 32.15, *p* < 0.001), Absolute Distance (*χ*(1) = 85.34, *p* < 0.001), Time (*χ*(1) = 19.50, *p* < 0.001), and the interaction between Absolute Distance and Time (*χ*(1) = 14.17, *p* < 0.001). As shown in Fig. 6, RTs were slower in Experiment 3, and increased when the expression was closer to the subjective middle; RTs for future expressions were faster than for past expressions, and the slope of the Distance effect was steeper for past than for future expressions.

**Table 4:** Comparisons of fit models

Model equation	Fixed effects	Model fit				
		Df	AIC	BIC	LL	Deviance
RTmean ~ Exp * stmmAbs *Time + (1 + stmmAbs*Time|SubjectID)	Experiment + absolute Distance + time + experiment × absolute distance + experiment × time + absolute distance x time + experiment × absolute distance × time	23	24,514	24,642	− 12,234	24,468
RTmean ~ Exp + stmmAbs *Time + (1 + stmmAbs*Time|SubjectID)	Experiment + absolute distance + time + absolute distance × time	17	24,507	24,601	− 12,236	24,473

*AIC* Aikake Information Criterion, *BIC* Bayesian Information Criterion, *LL* LogLikelihood, *Df* degrees of freedom, *RTmean* reaction times average, *Exp* Experiment (1–3), *stmmAbs* absolute distance, *Time* time (past vs. future), 1 + stmmAbs*Time|SubjectID random intercept and random slopes of absolute distance × time over participants

## Discussion

The purpose of our study was to provide a conclusive answer to the question of whether the mental representation of time is continuous or categorical in an explicit time judgment task. To do so, we asked participants to place 20 Italian temporal expressions on a line and then used the position of each term on the line to assess both the Distance and STEARC effects in three experiments with increasing saliency of the lateral spatial dimension. The results showed that Italian speakers placed the 20 temporal expressions in an ordered pattern from left to right along the line. Even if the temporal meanings were not perfectly spaced at regular intervals along the line, they covered a broad spectrum and could be used as a continuous predictor. Then, to verify our hypothesis, we contrasted the predictive ability of the continuous vs. the categorical predictor of the RT difference between the right and the left hands. The results were very clear. Responses with the right hand were faster for future than past terms compared to responses of the left hand, confirming the STEARC effect (see Bonato et al., (2012), for a review). Crucially, the model that only included the categorical predictor Time (past vs. future) showed a better fit than the model including only the continuous Distance predictor. Moreover, when both predictors were included simultaneously in the regression, the continuous predictor made no contribution to model fit over and above that made by the categorical predictor. Therefore, the STEARC effect is categorical, and not continuous, in explicit time judgment tasks. However, the results also showed a Distance effect, which is continuous by its own nature. The fact that the very same responses that reveal a categorical STEARC effect when the latencies of the right hand are subtracted from the left hand also reveal a continuous Distance effect when considered independently of the responding hand suggests that both effects reveal the characteristics of a single underlying representation. If this representation was categorical, it could not support a continuous effect such as the Distance effect. However, a continuous representation can be used categorically if a criterium is set and used to make binary decisions. Therefore, the present findings can be best interpreted as the categorical use of a continuous mental representation.

This result runs contrary to the findings by Ding et al. (2015), who observed a greater STEARC effect when people considered past events that were distant than when they were closer to the present. The present data show that the magnitude of the STEARC effect does not depend on the temporal distance from the present, neither toward the past nor toward the future. So far, the reasons for this discrepancy remain unclear. The temporal intervals used by Ding et al., (2015; a day and a year) were well within the range used in the present experiments. Other obvious differences are the language (Chinese vs. Italian) and the cultures (Western vs. East Asian). However, we cannot think of any reason why these factors could produce a continuous SNARC effect in China, but a categorical one in Italy.

The present results are, on the contrary, perfectly consistent with the study by Gevers et al. (2006), who observed, in the numerical domain, that the size of the SNARC effect varied continuously with number magnitude in a parity judgment task (implicit task), but was categorical in a magnitude comparison task (explicit task), suggesting an underlying continuous representation that can be used categorically when the task imposes explicit magnitude comparisons with a criterion. For a perfect parallelism between the domains of number and time, the STEARC effect should take a continuous form in implicit temporal tasks, as reported by Lakens et al. (2011), and by Gevers et al., (2003, 2004) in implicit order tasks. To test this final prediction, future research should replicate these studies and expressly assess the categorical vs. continuous form of the effect. In any case, the present study demonstrated a further similarity between the SNARC and STEARC effects, suggesting that similar mapping mechanisms are used to mentally represent the abstract concepts of time and number.

The present data also reveal interesting aspects regarding the continuous underlying representation of time, as they confirm that the Distance effect is not symmetrical toward the past and the future: latencies decreased with distance from the present at a greater rate toward the past than toward the future. In other words, psychological distance toward the future grew more slowly than toward the past. This was so in spite of the fact that most future references were placed further away from the subjective center than past references in the Time-to-Position task. Moreover, latencies for future temporal references were overall faster than those for the past. It is tempting to interpret this pattern as the result of cultural effects on temporal cognition, as Western cultures pay more attention to the future than to the past (Callizo-Romero et al., 2020; de la Fuente et al., 2014); they feel that the future is closer than the past (Caruso et al., 2013), give it a greater emotional and economic valuation (Caruso et al., 2008; Molouki et al., 2019), and feel more continuity with their future than past selves (Quoidbach et al., 2013). Some studies with Chinese participants (but not all) have found the opposite pattern (Guo et al., 2012; Guo & Spina, 2019; Ji et al., 2019; see Gao (2016), for a balanced review). Lacking a cross-cultural comparison, this possibility must remain an interesting speculation for now. Additionally, this asymmetrical pattern could be explained by the age of the participants. According to de la Fuente et al., (2014); see also Bylund et al., (2020), older participants focus less on the future and more on the past than younger participants. Since all our participants were university students, their young age may have influenced their future temporal horizons. Further studies are needed to investigate this issue.

An additional, and surprising finding of the present study is that Experiment 3, using mirror-reversed text, failed to replicate the findings by Casasanto and Bottini (2014). These authors found that a short exposure to mirror-reversed instructions and stimuli reversed the direction of participants’ MTL in the second block of their study (after 48 trials), which they explained in terms of representational flexibility. In our data, the STEARC effect did not vary across experiments. This was so even when only the second half of all three experiments was analyzed by means of ANOVA (*F* < 1). What is the cause of this failure of replication? There were several procedural differences between Casasanto and Bottini (2014) and the present Experiment 3: first, they presented the instructions in mirror-reversed text, whereas we did not; second, they used temporal sentences with a fixed structure (e.g., “a month before”, “a year before”, “a month after”, “a year after”), whereas we used adverbial temporal expressions; third, they presented each stimulus only once per block, whereas we presented them nine times; fourth, they presented all materials centered on the screen, whereas we presented them in left, center, and right positions. Even though we did not mirror-reverse the instructions, we provided much more practice with the experimental stimuli during the experiment (360 versus 86 trials), so we doubt that insufficient exposure to mirror text was an issue. It does not seem likely to us that the differences in materials and screen presentation could block the reversal of the effect. We think that the best candidate for the differential findings is stimulus repetition: by repeating the expressions nine times, participants may have adopted a holistic stimulus recognition strategy. By doing so, they would identify the expression and access its temporal meaning without actually proceeding from right to left over the letters of the stimulus. Thus, they were not having a right-to-left sensory-motor experience that could reverse the directionality of the MTL. If we are correct, future studies on the effect of mirror-reversing text on temporal cognition should be careful to avoid repeating the materials up to the point of making them recognizable as wholes. It is worth noting that ours is not the only study which has failed to replicate Casasanto and Bottini (2014). Yang et al. (2020) showed that in Japanese speakers, who are used to reading both horizontally and vertically, mirror reading reversed the vertical, but not the horizontal time line. These authors suggest that representational flexibility may depend on the relative practice using a script with a given orientation. Although this possibility does not apply to the present study (our participants only had practice with a left-to-right script), Yang et al.’s (2020) study suggests that we still do not know all the factors that allow for or prevent reversals of the MTL as a result of mirror reading.

In conclusion, the present study located the position of 20 temporal abstract concepts on a spatial line, from the far past to the far future, and used it to predict the size of the STEARC effect and the Distance effect in an explicit temporal judgment task. The STEARC effect showed a categorical pattern, but the Distance effect suggested that the underlying mental representation of time is continuous. This continuous MTL can be used categorically when the task requires a fixed criterium to be set to support temporal decision-making.
